# Black psychiatrists’ experience of discrimination and related behaviours in the workplace: UK survey

**DOI:** 10.1192/bjb.2025.7

**Published:** 2026-02

**Authors:** Jade Hombo, Lovita Owusu-Mensah, Martin Orrell, Mona-Lisa Kwentoh

**Affiliations:** 1 Faculty of Medicine and Health Sciences, University of Nottingham, Nottingham, UK; 2 Institute of Mental Health, University of Nottingham, Nottingham, UK; 3 Association of Black Psychiatrists United Kingdom, London, UK

**Keywords:** Stigma and discrimination, education and training, social functioning, qualitative research, ethics

## Abstract

**Aims and method:**

In the UK, Black doctors experience higher levels of discrimination, bullying and harassment compared with other doctors. This study aims to explore the impact of this on perceived well-being and mental health. A UK survey of 109 Black psychiatrists asked about racism, othering, microaggressions, bullying and harassment, plus any links to career progression or mental well-being.

**Results:**

Sixty-three survey participants (57.8%) had faced workplace microaggressions, 44 (40.4%) had experienced workplace bullying and 41 (37.6%) had faced workplace harassment. Forty-seven (43.1%) participants reported a detrimental impact on their mental health, with 35 (32.1%) considering quitting and 24 (22%) reporting a poorer work performance.

**Clinical implications:**

These experiences are unacceptable and can be traumatic. The impact of racism and discrimination can also undermine effective service delivery. Barriers to reporting can prolong mistreatment and deter professional aspirations among Black psychiatrists. Collective action is needed to drastically improve the workplace environment, including the widespread institutional adoption of an anti-discriminatory stance.

The UK's National Health Service (NHS) has benefited from diversity in its workforce, with international recruitment campaigns attracting professionals worldwide.^1^ Of the 45% of NHS doctors from Black, Asian and minority ethnic (BAME) backgrounds, 5.9% are Black.^2^ Thirty-nine per cent of the Royal College of Psychiatrists’ (RCPsych's) members are BAME, 6% are Black.^3^

Racial disparities in discrimination reported by NHS staff are evident: in a 2019 survey, 15.3% of BAME staff reported experiencing discrimination from other colleagues, compared with 6.4% of White staff.^4^

Bullying is defined as repeated practices aimed at victimising employees.^5^ The 2019 NHS Workforce Race Equality Standard (WRES) survey reported that 28.6% of BAME staff experienced harassment, bullying or abuse from other staff in a year, compared with 23.6% of White staff.^6^ In the UK, Black doctors face more racism from patients and staff than their minority ethnic counterparts.^7^

Microaggressions, a ‘covert’ form of racism, can reinforce negative stereotypes.^8^ For example, recurrent questioning of a clinician's competency often leads to feeling invalidated.^8^ Black and Asian doctors are more than 10 times more likely to report having their clinical work unfairly scrutinised compared with their White counterparts.^9^

High numbers of Black and Asian doctors report workplace social exclusion, with evidence that this can affect learning and, potentially, success in postgraduate examinations.^9^ This may reflect ‘othering’, which singles out individuals who differ from the norm.^10^

Despite WRES setting out quality improvement indicators, significant inequalities still exist.^6^ The RCPsych has highlighted the negative experiences of BAME doctors.^3^ However, specific discrimination against Black psychiatrists remains under-explored.

## Aims

The aim of this study was to investigate the experience of Black psychiatrists working in the UK healthcare system regarding discriminatory behaviours such as racism, microaggressions, bullying and harassment and to examine the impact of these experiences on their perceived well-being.

## Method

### Study design

An anonymous web-based survey was designed by a team of psychiatrists (M.-L.K., Dr Chinwe Obinwa and Dr Oluwaseun Oluwaranti) who are members of the Association of Black Psychiatrists UK (ABP-UK). The web-based survey was open to any psychiatrist who identified as Black and working in the UK. It was disseminated via the 70 members of the ABP-UK, who were encouraged to share it with their colleagues. Responses were invited from participants at varying levels of training in the UK. This included consultants, trainees, specialist and associate specialist (SAS) doctors and other related grades of doctor. A virtual snowball sampling technique was utilised in sending questionnaires to potential participants with the opportunity to respond within a 6-week period, from 14 December 2020 to 31 January 2021. Participants were asked about their experiences of discrimination in the workplace, including racism, microaggressions and othering. Participants were also asked to comment on its impact on career progression and their mental well-being. Some questions invited answers in the form of free-text comments to illustrate a broad range of views and experiences.

Ethical approval was not needed for this study as it involved a web-based survey with anonymised data, no identifiable personal information and informed consent obtained from staff participants.

### Data analysis

Data from the quantitative aspects of the survey were summarised using frequencies and percentages. Qualitative data from the free-text comments were analysed using thematic analysis based on the Braun & Clarke six-step process.^11^ After becoming familiar with the data-set (free-text comments), two reviewers (J.H. and L.O.-M.) analysed and collated codes for potential themes. These codes were reviewed separately by an experienced reviewer (M.O.). This helped to eliminate code bias and improve rigour. The codes, code definitions, themes and sub-themes were agreed by all parties.

## Results

### Demographics of survey participants

The survey was completed by 109 people, with 54 (49.5%) male and 55 (50.5%) female respondents. In terms of professional level, the respondents included 40 (36.7%) consultants, 33 (30.3%) SAS doctors, 32 (29.4%) trainees and 4 (3.7%) other doctors.

### Bullying and harassment

In total, 44 participants (40.4%) had experienced workplace bullying. Only 19 (43%) of them had reported these incidents and 8 (42%) of these of cases were addressed. In total, 41 (37.6%) had experienced workplace harassment, 17 (41.5%) of whom reported these incidents; only 8 (47%) of the reported cases were addressed. Participants provided 65 comments relating to barriers to reporting bullying and harassment, with two main themes emerging as ‘not feeling safe’ and ‘system failure’ (Table 1).

**Table 1:** Barriers to reporting bullying and harassment

Themes	Sub-themes	Comments
Not feeling safe (24 comments)	Impact on career	‘I was told it would hurt my future’ ‘Fear of being unemployed and having my visa withdrawn’
	Fear of repercussions	‘I may even be targeted’ ‘Fear of a witch hunt’
	Lack of support	‘Lack of support and fear of victimisation’
System failure (41 comments)	No action	‘Lack of confidence in the system to address my concerns’ ‘I reported it but it wasn't actioned’ ‘Predecessors from ethnic minority experienced same and nothing was done’ ‘Concern it won't be addressed, behaviour would increase in intensity and frequency. Further alienation from other colleagues who were aware of the behaviour’
	Not knowing how to report	‘Did not know whom to report it to’ ‘Nothing good would happen, stigma to me, no clear process regarding discrimination itself’

### Racism and microaggressions

In total, 63 (57.8%) of the survey participants reported experiencing microaggression in the workplace; 25 (39.7%) of those reported questioning this behaviour and 16 (64%) of those who questioned the behaviour felt supported by their colleagues/line managers to do so. Othering was experienced in the workplace by 43 (39.4%) participants and was challenged by 9 (20.9%), with only 7 (77.8%) of those who challenged it feeling supported by colleagues/line managers in doing so.

### Impact on work and mental well-being

In total, 54 (49.5%) participants reported that they enjoyed going to work. In total, 47 (43.1%) participants reported that their experiences of bullying and harassment, racism and microaggressions had negatively affected their mental health, 24 (22%) participants indicated that these experiences had an impact on their work performance and 35 (32.1%) stated that they had contemplated quitting their employment because of discriminatory treatment.

### Thematic analysis

#### Barriers to reporting bullying and harassment

There were 65 comments regarding barriers to reporting bullying and harassment. The main themes identified were participants not feeling safe and a perception of being failed by the system. The 24 comments related to participants not feeling safe could be linked to the sub-themes of impact on career, fear of repercussions and lack of support; the 41 comments related to system failure included participants not knowing how to report incidents or that when they did the system failed to act on the report (Table 1).

#### Experiences with discriminatory behaviours

There were 117 comments relating to discriminatory behaviours. The main themes that emerged were othering and belittling (often as microaggressions), questioning competency and workload inequality (Table 2). Participants mentioned that many inappropriate comments were made regarding their appearance, with criticism of their cultural expression of clothing and hair. Remarks about their ethnicity, name, accent and country of origin were also noted, making them feel uncomfortable.

**Table 2:** Experiences with discriminatory behaviours

Themes	Sub-themes	Comments
Othering and belittling (75 comments)	Comments on appearance	‘My clothes were described as pyjamas’ ‘Saying my hair styles [change] too often, it must cost me a fortune’
	Comments relating to culture	‘Being called Caribbean’ ‘Being called intimidating and scary’ ‘Not being introduced at meetings’ ‘Never getting my name right’
Questioning competency (27 comments)		‘Snide remarks from colleagues’ ‘Constantly questioning my clinical plans’ ‘Questioned about my education, qualifications, clinical expertise when non-Black colleagues weren't’ ‘Doubting my clinical judgement based on West African training’ ‘Compliments, like you do it so well as compared to other Black doctors’
Workload inequality (15 comments)		‘Coerced to work outside my job plan’
		‘Interrupted rudely while talking in a meeting'
		‘Ignoring my comments in favour of those from a White colleague’
		‘Working twice as hard for less praise’
		‘Being expected to work past breaking point’

The 27 comments involving questioning the participants’ competency related to their academic background, qualifications and clinical reasoning. Participants indicated that comments from colleagues had suggested the rigour of their clinical decision-making was surprising when compared with other Black peers they had encountered.

In the 15 comments related to workload inequalities, several participants stated they were expected and encouraged to work outside of their agreed job plan. Participants also reported having a higher workload compared with their peers.

## Discussion

This study aimed to examine Black psychiatrists’ experience of discrimination and related behaviours in the workplace. Overall, the findings show that Black psychiatrists in the UK experience racism, microaggressions, bullying and harassment. These experiences had a deleterious impact on their mental well-being and ability to care for patients, and led to contemplation of role changes. Additionally, a considerable proportion of Black psychiatrists reported facing barriers when attempting to report these challenging experiences.

### Bullying and harassment

The finding that less than half of the incidents of bullying and harassment were being reported, with even fewer cases being addressed, aligns with observations in other reports.^12,13^ The consequences of bullying and harassment are grave, with potential psychological implications such as work-related depression, psychosomatic symptoms, stress, reduced sleep quality and impairments in job performance and satisfaction.^5,14^ Workplace dissatisfaction can prompt healthcare professionals to leave their roles.^15^ This is likely applicable to Black psychiatrists, as survey participants reported that they had contemplated quitting their employment because of discriminatory experiences at work. Recent years have witnessed a staff retention crisis in the NHS which has been attributed to poor working conditions, including bullying and harassment.^15^ Retaining and supporting staff is a key property outlined in the NHS long-term plan.^16,17^

### Barriers to reporting bullying and harassment

The barriers to reporting incidents of bullying and harassment included not feeling safe and system failure. Participants felt a lack of support or believed that reporting incidents would have an impact on their careers or hurt their future, including professional development and career success. This could pose a threat to the diversity of thought among clinical leaders, which is pertinent to healthcare outcomes, particularly as doctors should be representative of the communities they serve.^18,19^ Diversity among healthcare professionals would ensure a better understanding of patients’ care needs, accessibility and the challenge of healthcare inequalities.^20^

The fear of job loss because of reporting experiences of bullying or harassment potentially silences Black doctors. This can lead to a cycle of harassment or bullying as racism continues. Concerns about system failure imply that the policies and procedures put in place to protect psychiatrists at work have failed them. If not addressed, these toxic work environments threaten diversity in the NHS, directly affecting patient care.^19^ Furthermore, participants’ comments showed that the system was ineffective, as many people did not know how to access or use it. In some cases, line managers were perpetrators, which indicates that those who are part of the ‘system’ to safeguard psychiatrists may be failing to do so and likely make matters worse through their own actions.

### Discriminatory behaviour

Almost 60% of participants experienced microaggressions in the workplace. Microaggressions, which involve othering and belittling, can often be used to propagate racism. This includes instances where participants were called ‘intimidating and scary’, reflecting prejudices that Black people may be perceived as ‘angry or aggressive’. This could foster an environment for exclusion and marginalisation. Some othering experiences in the workplace occur in the form of seemingly inquisitive secondary questions such as ‘where you are really from?’, particularly when an individual has clearly indicated being British.^8^

Comments about their appearance, including remarks on clothes and hair, played a significant role in making participants feel alienated. This could suggest anti-Black hair sentiment occurring in the workplace. Black hair discrimination has long existed in the workplace, with some hairstyles criticised as being unprofessional.^21–24^ Some participants reported behaviours such as ‘hair touching’ by colleagues, which suggests the constant monitoring of Black hair. This could be deemed intrusive, potentially harming the identity of Black psychiatrists and their cultural expression. Comments highlight how Black doctors were not encouraged to celebrate their differences and individuality, which exacerbates the feeling of being deemed as ‘other’.

Comments also show that participants were misidentified in the workplace, for example being ‘mixed up with other female doctors’. Although research suggests that people identify faces from their own race better,^25^ one participant reported colleagues getting their ‘name wrong’, despite working with them for many years. This may suggest a lack of willingness to acknowledge individuality among Black doctors, despite close working relationships that would better support familiarity. This experience in the healthcare system could be considered a microcosm for society, where Black people are likely grouped as ‘one’. Misidentification could carry the potential for missed opportunities for career progression and feeling less valued or invisible at work.

### Workload inequality

One of the sub-themes that emerged was workload inequalities, which may be both quantitative and qualitative in nature.^26^ Survey participants describe being ‘coerced outside their job plan’, which could indicate a lack of control and autonomy and pressure from others to work past their limit (Table 2). This excessive workload could lead to burnout, which would affect clinical decisions. Working outside a job plan could be considered a form of exploitation, especially without additional remuneration.

These findings seem to echo the widely reported concept that Black people must work twice as hard to achieve similar levels of success as their White counterparts.^27^ Senior figures such as mentors and parental figures in Black communities have also been known to encourage relatives or mentees to work ‘twice as hard’ as their White peers to be considered intelligent or talented.^28^

### Questioning competency

Comments showing that Black psychiatrists’ competency was questioned are similar to results of previous studies.^9^ This highlights the disproportionate scrutiny of Black doctors’ clinical ability and suggests that they may not be celebrated in the same way as their White counterparts. Commenting on education, qualifications and clinical expertise in front of others is likely to undermine perceptions of competency and fuel feelings of mistrust. Evidence suggests that Black people perceive being subjected to a professional standard of ‘Whiteness’ in the workplace.^29^ This has led to a call for a rethink on the value systems that drive the professional and organisational discourse in workplaces.^29^

### Strengths and limitations

We used virtual snowball sampling because of its ability to improve response rates, especially when pertaining to certain participant demographics and qualitative exploration of sensitive topics.^30^ However, a disadvantage is that because of the use of snowball sampling we were not able to provide an estimated response rate. The sample for this study was psychiatrists who identified as Black working in the UK. As such, it likely represents a broad range of ages, gender, perspectives and experience. The use of snowball sampling may also increase the risk of bias. However, there were attempts to mitigate this by anonymising the survey tool, independent data analysis, online data collection and extraction.

It is unclear what proportion of responses were from the members of Association of Black Psychiatrists UK. This is a professional association that aims to provide a safe space for networking, learning, development and support. Some participants may therefore have felt more comfortable in sharing their difficult workplace experiences, thereby improving the yield of responses to the sensitive issues being raised.

The geographical representation of workplace experiences across the UK was not explored in the survey as participants were not asked what area they worked in. Furthermore, this paper is based on descriptive statistics. Bivariate statistics would have further explored whether or not there was a relationship between variables such as experience of discrimination and gender.

### Implications

This study has highlighted under-reporting of discriminatory behaviours against Black psychiatrists in the workplace, and future work should seek to critically examine the organisational and structural hurdles that exist in reporting discrimination and related behaviours. Taking an anti-racism stance must be a deliberate decision. The Royal College of Psychiatrists’ guidance for employers on tackling racism in the workplace is a call to action, encouraging organisations to address discrimination at a strategic and systemic level.^31^ Its 15 recommended actions are grounded in expert evidence and their adoption could assist organisations in creating more equitable workplaces, which would be beneficial to current and future employees.^31^ This, in turn, could lead to effectively addressing adverse workplace experiences, fostering a positive culture for psychological safety, facilitating necessary learning to deconstruct harmful behaviours and implementing monitoring of progress to address these issues.^29^

## Data Availability

The data that support the findings of this study are available on request from M.-L.K. (mkwentoh@nhs.net).
